# 2-[(2,6-Diisopropyl­phen­yl)imino­meth­yl]-4-iodo­phenol

**DOI:** 10.1107/S1600536812023653

**Published:** 2012-05-31

**Authors:** P. Balamurugan, K. Kanmani Raja, I. Mohammed Bilal, G. Chakkaravarthi, G. Rajagopal

**Affiliations:** a Department of Chemistry, Government Arts College (Men), Nandanam, Chennai 600 035, India; b Department of Chemistry, Government Thirumagal Mills College, Gudiyattam 632 604, India; c Department of Chemistry, B.S. Abdur Rahman University, Vandalur, Chennai 600 049, India; d Department of Physics, CPCL Polytechnic College, Chennai 600 068, India; e Department of Chemistry, Government Arts College, Melur 625 106, India

## Abstract

The asymmetric unit of title compound, C_19_H_22_INO, contains two independent mol­ecules. Classical intra­molecular O—H⋯N hydrogen bonds stabilize the mol­ecular structures. The crystal structure is stabilized by weak inter­molecular C—H⋯π and π–π [centroid–centroid = 3.8622 (18) Å] inter­actions. In both mol­ecules, the aromatic rings are nearly perpendicular to each other [dihedral angles = 84.26 (17) and 86.69 (15)°].

## Related literature

For the biological activity of Schiff base ligands, see: Santos *et al.* (2001 ▶). For related strucutures, see: Raja *et al.* (2008 ▶); Lin *et al.* (2005 ▶).
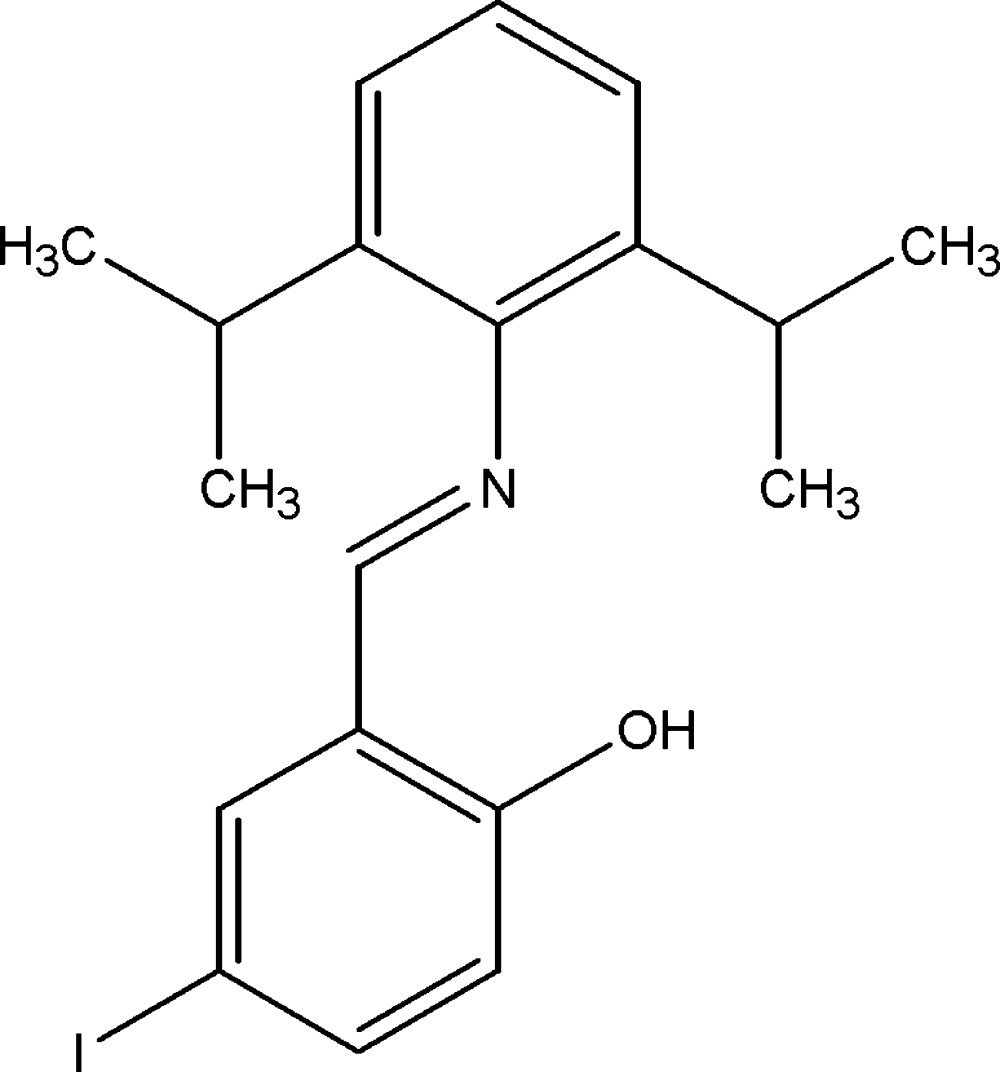


## Experimental

### 

#### Crystal data

C_19_H_22_INO
*M* *_r_* = 407.28
Triclinic, 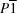

*a* = 5.9891 (2) Å
*b* = 12.4270 (5) Å
*c* = 25.8832 (10) Å
α = 83.065 (2)°
β = 84.860 (3)°
γ = 76.408 (2)°
*V* = 1855.00 (12) Å^3^
*Z* = 4
Mo *K*α radiation
μ = 1.73 mm^−1^
*T* = 295 K
0.26 × 0.24 × 0.20 mm

#### Data collection

Bruker Kappa APEXII CCD diffractometer
Absorption correction: multi-scan (*SADABS*; Sheldrick, 1996 ▶) *T*_min_ = 0.662, *T*_max_ = 0.724
45699 measured reflections
10505 independent reflections
7252 reflections with *I* > 2σ(*I*)
*R*_int_ = 0.025

#### Refinement

*R*[*F* ^2^ > 2σ(*F* ^2^)] = 0.039
*wR*(*F* ^2^) = 0.111
*S* = 1.01
10505 reflections
407 parameters
H-atom parameters constrained
Δρ_max_ = 0.91 e Å^−3^
Δρ_min_ = −0.65 e Å^−3^

### 

Data collection: *APEX2* (Bruker, 2004 ▶); cell refinement: *SAINT* (Bruker, 2004 ▶); data reduction: *SAINT*; program(s) used to solve structure: *SHELXS97* (Sheldrick, 2008 ▶); program(s) used to refine structure: *SHELXL97* (Sheldrick, 2008 ▶); molecular graphics: *PLATON* (Spek, 2009 ▶); software used to prepare material for publication: *SHELXL97*.

## Supplementary Material

Crystal structure: contains datablock(s) global, I. DOI: 10.1107/S1600536812023653/rk2360sup1.cif

Structure factors: contains datablock(s) I. DOI: 10.1107/S1600536812023653/rk2360Isup2.hkl

Supplementary material file. DOI: 10.1107/S1600536812023653/rk2360Isup3.cml

Additional supplementary materials:  crystallographic information; 3D view; checkCIF report

## Figures and Tables

**Table 1:** Hydrogen-bond geometry (Å, °) *Cg*2 is the centroid of the C8–C13 ring.

*D*—H⋯*A*	*D*—H	H⋯*A*	*D*⋯*A*	*D*—H⋯*A*
O1—H1⋯N1	0.82	1.88	2.606 (3)	147
O2—H2*A*⋯N2	0.82	1.91	2.617 (3)	143
C16—H16*A*⋯*Cg*2^i^	0.96	2.91	3.785 (5)	153

Symmetry code: (i) 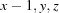.

**Fig. 1.**
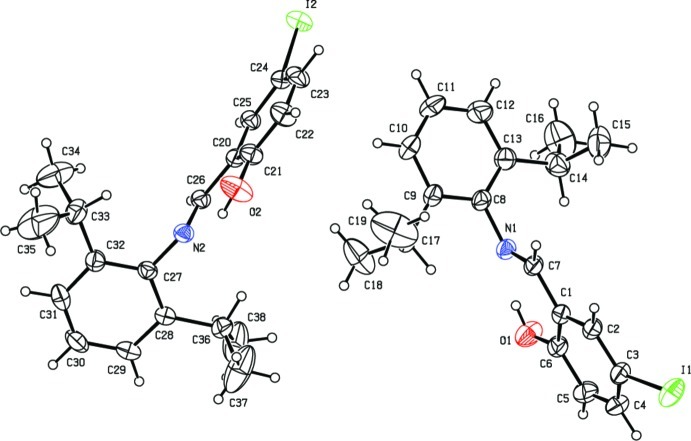
The molecular structure of title compound with the atom labels. Displacement ellipsoids are drawn at 30% probability level. H atoms are presented as a small spheres of arbitrary radius.

**Fig. 2.**
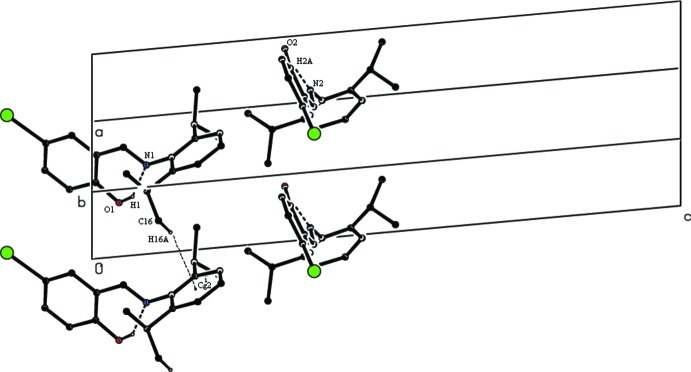
The intramolecular O–H···N hydrogen bonds and intermolecular C–H···π interaction (dashed lines) in crystal structure of title compound.

## supplementary crystallographic information

### Comment

Schiff base derivatives exhibit antibacterial, antitumor and antitoxic activities (Santos *et al.*, 2001). The asymmetric unit of the title compound I, (Fig. 1), contains two independent molecules. The geometric parameters in I are comparable with the similar reported structures (Lin *et al.*, 2005; Raja *et al.*, 2008). The dihedral angles between the benzene rings (C1-C6) and (C8-C13) & (C20-C25) and (C27-C32) are 84.26 (17)° and 86.69 (15)°. Further, both molecules adopts anti-periplanar (C1-C7-N1-C8 = 177.2 (2)° and C20-C26-N2-C27 = 175.8 (2)°) conformation about C═N bond.

The molecular structure is stabilized by weak intramolecular O–H···N hydrogen bonds and the crystal structure exhibit weak intermolecular C–H···π (Cg2^i^) (Table 1, Fig. 2) and π–π interactions (Cg1···Cg1^ii^) with distance 3.8622 (18)Å. Cg1 is the centroid of (C1-C6) ring; Cg2 is the centroid of (C8-C13) ring. Symmetry codes: (i) *x*-1, *y*, *z*; (ii) 1-*x*, 1-*y*, -*z*.

### Experimental

An ethanolic solution (10 ml) of 2,6-diisopropylaniline (2 mmol) was stirred in a round bottom flask followed by drop wise addition of ethanolic solution (10 ml) of 5-iodosalicylaldehyde (2 mmol). The reaction mixture was then refluxed for 3 h and upon cooling to 273 K. A yellow solid precipitate from the reaction mixture was filtered out, washed with ice cold ethanol and dried over anhydrous CaCl_2_. Single crystals of good diffraction quality were obtained by the recrystallization of compound from ethanol solution by slow evaporation. Yield: 70 %.

### Refinement

The H atoms were positioned geometrically with C–H = 0.93-0.98Å and O–H = 0.82Å, and allowed to ride on their parent atoms, with *U*_iso_(H) = 1.5 *U*_eq_(O) (or) 1.2*U*_eq_(C) (or) 1.5*U*_eq_(C_methyl_).

### Crystal data

**Table:**

C_19_H_22_INO	*Z* = 4
*M**_r_* = 407.28	*F*(000) = 816
Triclinic, *P*1	*D*_x_ = 1.458 Mg m^−^^3^
Hall symbol: -P 1	Mo *K*α radiation, λ = 0.71073 Å
*a* = 5.9891 (2) Å	Cell parameters from 10508 reflections
*b* = 12.4270 (5) Å	θ = 0.8–29.8°
*c* = 25.8832 (10) Å	µ = 1.73 mm^−^^1^
α = 83.065 (2)°	*T* = 295 K
β = 84.860 (3)°	Prism, yellow
γ = 76.408 (2)°	0.26 × 0.24 × 0.20 mm
*V* = 1855.00 (12) Å^3^	

### Data collection

**Table:**

Bruker Kappa APEXII CCD diffractometer	10505 independent reflections
Radiation source: fine-focus sealed tube	7252 reflections with *I* > 2σ(*I*)
Graphite monochromator	*R*_int_ = 0.025
ω and φ scans	θ_max_ = 29.8°, θ_min_ = 0.8°
Absorption correction: multi-scan (*SADABS*; Sheldrick, 1996)	*h* = −8→8
*T*_min_ = 0.662, *T*_max_ = 0.724	*k* = −17→17
45699 measured reflections	*l* = −36→36

### Refinement

**Table:**

Refinement on *F*^2^	Primary atom site location: structure-invariant direct methods
Least-squares matrix: full	Secondary atom site location: difference Fourier map
*R*[*F*^2^ > 2σ(*F*^2^)] = 0.039	Hydrogen site location: inferred from neighbouring sites
*wR*(*F*^2^) = 0.111	H-atom parameters constrained
*S* = 1.01	*w* = 1/[σ^2^(*F*_o_^2^) + (0.0464*P*)^2^ + 1.3528*P*] where *P* = (*F*_o_^2^ + 2*F*_c_^2^)/3
10505 reflections	(Δ/σ)_max_ < 0.001
407 parameters	Δρ_max_ = 0.91 e Å^−^^3^
0 restraints	Δρ_min_ = −0.65 e Å^−^^3^

### Special details

**Table:**

Geometry. All s.u.'s (except the s.u. in the dihedral angle between two l.s. planes) are estimated using the full covariance matrix. The cell s.u.'s are taken into account individually in the estimation of s.u.'s in distances, angles and torsion angles; correlations between s.u.'s in cell parameters are only used when they are defined by crystal symmetry. An approximate (isotropic) treatment of cell s.u.'s is used for estimating s.u.'s involving l.s. planes.
Refinement. Refinement of *F*^2^ against ALL reflections. The weighted *R*-factor *wR* and goodness of fit *S* are based on *F*^2^, conventional *R*-factors *R* are based on *F*, with *F* set to zero for negative *F*^2^. The threshold expression of *F*^2^ > σ(*F*^2^) is used only for calculating *R*-factors(gt) *etc*. and is not relevant to the choice of reflections for refinement. *R*-factors based on *F*^2^ are statistically about twice as large as those based on *F*, and *R*-factors based on ALL data will be even larger.

### Fractional atomic coordinates and isotropic or equivalent isotropic displacement parameters (Å^2^)

**Table:**

	*x*	*y*	*z*	*U*_iso_*/*U*_eq_	
C1	0.4118 (5)	0.6977 (2)	0.00843 (10)	0.0464 (6)	
C2	0.5675 (5)	0.7050 (2)	−0.03417 (10)	0.0522 (6)	
H2	0.6832	0.7430	−0.0330	0.063*	
C3	0.5509 (6)	0.6561 (3)	−0.07792 (11)	0.0582 (7)	
C4	0.3847 (7)	0.5972 (3)	−0.07979 (13)	0.0668 (9)	
H4	0.3753	0.5643	−0.1097	0.080*	
C5	0.2343 (7)	0.5870 (3)	−0.03797 (14)	0.0685 (9)	
H5	0.1248	0.5454	−0.0392	0.082*	
C6	0.2420 (5)	0.6380 (2)	0.00656 (11)	0.0544 (7)	
C7	0.4231 (5)	0.7547 (2)	0.05361 (10)	0.0475 (6)	
H7	0.5400	0.7923	0.0539	0.057*	
C8	0.2887 (5)	0.8156 (2)	0.13537 (10)	0.0472 (6)	
C9	0.4337 (6)	0.7673 (3)	0.17544 (11)	0.0563 (7)	
C10	0.4269 (7)	0.8276 (3)	0.21734 (12)	0.0748 (10)	
H10	0.5214	0.7975	0.2445	0.090*	
C11	0.2842 (8)	0.9303 (4)	0.21954 (14)	0.0866 (12)	
H11	0.2825	0.9695	0.2481	0.104*	
C12	0.1436 (7)	0.9759 (3)	0.17999 (15)	0.0774 (10)	
H12	0.0490	1.0465	0.1819	0.093*	
C13	0.1385 (5)	0.9198 (3)	0.13727 (12)	0.0572 (7)	
C14	−0.0175 (6)	0.9713 (3)	0.09377 (16)	0.0722 (9)	
H14	−0.0246	0.9119	0.0727	0.087*	
C15	0.0816 (9)	1.0567 (4)	0.05887 (18)	0.1021 (15)	
H15A	0.0918	1.1159	0.0786	0.153*	
H15B	−0.0162	1.0862	0.0307	0.153*	
H15C	0.2324	1.0225	0.0451	0.153*	
C16	−0.2637 (8)	1.0227 (5)	0.1132 (2)	0.1217 (19)	
H16A	−0.3289	0.9666	0.1336	0.183*	
H16B	−0.3546	1.0528	0.0839	0.183*	
H16C	−0.2623	1.0811	0.1342	0.183*	
C17	0.5924 (6)	0.6533 (3)	0.17394 (12)	0.0661 (8)	
H17	0.5806	0.6279	0.1401	0.079*	
C18	0.5224 (10)	0.5710 (4)	0.2152 (2)	0.123 (2)	
H18A	0.5301	0.5942	0.2489	0.184*	
H18B	0.6244	0.4993	0.2125	0.184*	
H18C	0.3678	0.5662	0.2107	0.184*	
C19	0.8408 (9)	0.6554 (6)	0.1781 (3)	0.159 (3)	
H19A	0.8810	0.7129	0.1533	0.239*	
H19B	0.9373	0.5847	0.1710	0.239*	
H19C	0.8621	0.6701	0.2127	0.239*	
C20	0.7450 (4)	0.6051 (2)	0.36056 (10)	0.0411 (5)	
C21	0.9503 (5)	0.6272 (2)	0.33716 (12)	0.0531 (7)	
C22	0.9664 (6)	0.7363 (3)	0.32285 (15)	0.0663 (9)	
H22	1.1011	0.7511	0.3059	0.080*	
C23	0.7852 (6)	0.8224 (2)	0.33351 (13)	0.0601 (8)	
H23	0.7989	0.8954	0.3244	0.072*	
C24	0.5821 (5)	0.8019 (2)	0.35772 (11)	0.0474 (6)	
C25	0.5625 (5)	0.6937 (2)	0.37041 (10)	0.0460 (6)	
H25	0.4245	0.6796	0.3859	0.055*	
C26	0.7213 (4)	0.4913 (2)	0.37687 (10)	0.0427 (5)	
H26	0.5809	0.4800	0.3920	0.051*	
C27	0.8554 (4)	0.2998 (2)	0.39095 (10)	0.0421 (5)	
C28	0.7612 (5)	0.2388 (2)	0.36035 (11)	0.0510 (6)	
C29	0.7379 (6)	0.1340 (2)	0.38151 (14)	0.0651 (8)	
H29	0.6760	0.0914	0.3620	0.078*	
C30	0.8036 (6)	0.0917 (2)	0.43042 (15)	0.0694 (9)	
H30	0.7819	0.0220	0.4442	0.083*	
C31	0.9005 (6)	0.1515 (3)	0.45893 (14)	0.0660 (8)	
H31	0.9477	0.1212	0.4919	0.079*	
C32	0.9307 (5)	0.2568 (2)	0.43996 (11)	0.0496 (6)	
C33	1.0355 (6)	0.3225 (3)	0.47292 (13)	0.0663 (8)	
H33	1.0681	0.3866	0.4501	0.080*	
C34	0.8713 (10)	0.3675 (5)	0.5157 (2)	0.1163 (18)	
H34A	0.7312	0.4102	0.5014	0.174*	
H34B	0.9379	0.4144	0.5337	0.174*	
H34C	0.8387	0.3071	0.5397	0.174*	
C35	1.2620 (10)	0.2569 (6)	0.4929 (3)	0.160 (3)	
H35A	1.3437	0.3068	0.5042	0.240*	
H35B	1.3523	0.2178	0.4656	0.240*	
H35C	1.2339	0.2044	0.5217	0.240*	
C36	0.6849 (7)	0.2835 (3)	0.30611 (12)	0.0674 (9)	
H36	0.6893	0.3624	0.3008	0.081*	
C37	0.8456 (9)	0.2258 (7)	0.26518 (19)	0.157 (3)	
H37A	0.8594	0.1469	0.2719	0.236*	
H37B	0.9942	0.2421	0.2658	0.236*	
H37C	0.7864	0.2514	0.2315	0.236*	
C38	0.4445 (8)	0.2758 (6)	0.29912 (19)	0.121 (2)	
H38A	0.4439	0.2012	0.2931	0.181*	
H38B	0.3860	0.3262	0.2698	0.181*	
H38C	0.3492	0.2952	0.3300	0.181*	
I1	0.77120 (5)	0.67673 (3)	−0.144206 (9)	0.09004 (11)	
I2	0.31035 (4)	0.932551 (16)	0.377683 (10)	0.06823 (9)	
N1	0.2794 (4)	0.75482 (19)	0.09260 (8)	0.0477 (5)	
N2	0.8848 (4)	0.40812 (18)	0.37106 (9)	0.0449 (5)	
O1	0.0845 (5)	0.6295 (2)	0.04618 (9)	0.0760 (7)	
H1	0.1016	0.6665	0.0691	0.114*	
O2	1.1358 (4)	0.54504 (19)	0.32778 (13)	0.0823 (8)	
H2A	1.0987	0.4850	0.3325	0.124*	

### Atomic displacement parameters (Å^2^)

**Table:**

	*U*^11^	*U*^22^	*U*^33^	*U*^12^	*U*^13^	*U*^23^
C1	0.0554 (15)	0.0450 (14)	0.0400 (13)	−0.0130 (11)	−0.0087 (11)	−0.0022 (11)
C2	0.0640 (17)	0.0561 (16)	0.0394 (14)	−0.0191 (13)	−0.0049 (12)	−0.0041 (12)
C3	0.0696 (19)	0.0612 (18)	0.0408 (15)	−0.0069 (14)	−0.0054 (13)	−0.0080 (13)
C4	0.088 (2)	0.0637 (19)	0.0531 (18)	−0.0144 (17)	−0.0161 (17)	−0.0193 (15)
C5	0.083 (2)	0.068 (2)	0.066 (2)	−0.0317 (18)	−0.0164 (17)	−0.0153 (16)
C6	0.0649 (18)	0.0553 (16)	0.0476 (15)	−0.0216 (13)	−0.0102 (13)	−0.0029 (13)
C7	0.0572 (15)	0.0515 (15)	0.0386 (13)	−0.0212 (12)	−0.0055 (11)	−0.0040 (11)
C8	0.0554 (15)	0.0534 (15)	0.0355 (13)	−0.0209 (12)	0.0057 (11)	−0.0041 (11)
C9	0.0730 (19)	0.0602 (17)	0.0371 (14)	−0.0198 (15)	−0.0017 (13)	−0.0028 (12)
C10	0.104 (3)	0.083 (2)	0.0395 (16)	−0.022 (2)	−0.0094 (17)	−0.0111 (16)
C11	0.127 (4)	0.087 (3)	0.0495 (19)	−0.024 (3)	0.004 (2)	−0.0308 (19)
C12	0.096 (3)	0.066 (2)	0.067 (2)	−0.0123 (19)	0.009 (2)	−0.0187 (18)
C13	0.0635 (18)	0.0572 (17)	0.0516 (16)	−0.0179 (14)	0.0029 (13)	−0.0052 (13)
C14	0.068 (2)	0.062 (2)	0.084 (3)	−0.0083 (16)	−0.0141 (18)	−0.0043 (18)
C15	0.120 (4)	0.115 (4)	0.074 (3)	−0.045 (3)	−0.021 (3)	0.023 (3)
C16	0.073 (3)	0.126 (4)	0.149 (5)	−0.005 (3)	−0.009 (3)	0.019 (4)
C17	0.088 (2)	0.067 (2)	0.0421 (16)	−0.0137 (17)	−0.0141 (15)	−0.0018 (14)
C18	0.109 (4)	0.080 (3)	0.154 (5)	−0.008 (3)	0.025 (3)	0.033 (3)
C19	0.077 (3)	0.127 (5)	0.268 (9)	−0.019 (3)	0.047 (4)	−0.042 (5)
C20	0.0485 (13)	0.0399 (12)	0.0379 (12)	−0.0158 (10)	−0.0037 (10)	−0.0031 (10)
C21	0.0511 (15)	0.0474 (15)	0.0624 (18)	−0.0163 (12)	0.0034 (13)	−0.0066 (13)
C22	0.0600 (18)	0.0577 (18)	0.086 (2)	−0.0314 (15)	0.0084 (16)	0.0003 (16)
C23	0.0695 (19)	0.0413 (14)	0.075 (2)	−0.0264 (14)	−0.0055 (16)	0.0005 (14)
C24	0.0574 (15)	0.0394 (13)	0.0479 (15)	−0.0134 (11)	−0.0074 (12)	−0.0060 (11)
C25	0.0508 (14)	0.0412 (13)	0.0471 (14)	−0.0151 (11)	0.0001 (11)	−0.0030 (11)
C26	0.0472 (13)	0.0404 (13)	0.0423 (13)	−0.0152 (10)	0.0023 (10)	−0.0047 (10)
C27	0.0436 (13)	0.0358 (12)	0.0443 (14)	−0.0053 (10)	0.0024 (10)	−0.0053 (10)
C28	0.0642 (17)	0.0393 (13)	0.0507 (16)	−0.0123 (12)	−0.0058 (13)	−0.0067 (11)
C29	0.086 (2)	0.0378 (14)	0.075 (2)	−0.0168 (14)	−0.0072 (17)	−0.0114 (14)
C30	0.087 (2)	0.0340 (14)	0.082 (2)	−0.0088 (14)	−0.0054 (19)	0.0071 (15)
C31	0.073 (2)	0.0535 (17)	0.063 (2)	−0.0055 (15)	−0.0123 (16)	0.0161 (15)
C32	0.0494 (14)	0.0513 (15)	0.0453 (15)	−0.0071 (12)	−0.0018 (11)	−0.0027 (12)
C33	0.072 (2)	0.081 (2)	0.0519 (17)	−0.0277 (17)	−0.0118 (15)	−0.0035 (16)
C34	0.127 (4)	0.136 (4)	0.105 (4)	−0.054 (3)	0.027 (3)	−0.065 (3)
C35	0.106 (4)	0.204 (7)	0.181 (7)	0.002 (4)	−0.072 (4)	−0.085 (6)
C36	0.104 (3)	0.0578 (18)	0.0497 (17)	−0.0327 (18)	−0.0160 (17)	−0.0044 (14)
C37	0.084 (3)	0.300 (10)	0.063 (3)	−0.001 (4)	0.005 (2)	−0.019 (4)
C38	0.077 (3)	0.182 (6)	0.081 (3)	0.007 (3)	−0.018 (2)	0.014 (3)
I1	0.1026 (2)	0.1210 (2)	0.04660 (13)	−0.02447 (17)	0.00854 (12)	−0.02049 (13)
I2	0.07654 (15)	0.04092 (11)	0.08644 (17)	−0.00987 (9)	−0.00392 (11)	−0.01118 (10)
N1	0.0552 (13)	0.0516 (13)	0.0394 (11)	−0.0199 (10)	0.0001 (10)	−0.0041 (10)
N2	0.0503 (12)	0.0404 (11)	0.0451 (12)	−0.0140 (9)	0.0022 (9)	−0.0058 (9)
O1	0.0842 (16)	0.0992 (19)	0.0619 (14)	−0.0542 (15)	0.0054 (12)	−0.0181 (13)
O2	0.0569 (13)	0.0544 (13)	0.131 (2)	−0.0179 (10)	0.0303 (14)	−0.0085 (14)

### Geometric parameters (Å, º)

**Table:**

C1—C2	1.388 (4)	C20—C21	1.393 (4)
C1—C6	1.400 (4)	C20—C26	1.462 (3)
C1—C7	1.452 (4)	C21—O2	1.347 (4)
C2—C3	1.370 (4)	C21—C22	1.385 (4)
C2—H2	0.9300	C22—C23	1.367 (5)
C3—C4	1.373 (5)	C22—H22	0.9300
C3—I1	2.097 (3)	C23—C24	1.382 (4)
C4—C5	1.358 (5)	C23—H23	0.9300
C4—H4	0.9300	C24—C25	1.375 (4)
C5—C6	1.390 (4)	C24—I2	2.089 (3)
C5—H5	0.9300	C25—H25	0.9300
C6—O1	1.342 (4)	C26—N2	1.259 (3)
C7—N1	1.266 (3)	C26—H26	0.9300
C7—H7	0.9300	C27—C32	1.387 (4)
C8—C13	1.396 (4)	C27—C28	1.396 (4)
C8—C9	1.398 (4)	C27—N2	1.427 (3)
C8—N1	1.425 (3)	C28—C29	1.383 (4)
C9—C10	1.383 (4)	C28—C36	1.515 (4)
C9—C17	1.512 (5)	C29—C30	1.366 (5)
C10—C11	1.363 (6)	C29—H29	0.9300
C10—H10	0.9300	C30—C31	1.359 (5)
C11—C12	1.367 (6)	C30—H30	0.9300
C11—H11	0.9300	C31—C32	1.387 (4)
C12—C13	1.383 (5)	C31—H31	0.9300
C12—H12	0.9300	C32—C33	1.515 (4)
C13—C14	1.511 (5)	C33—C34	1.483 (6)
C14—C15	1.504 (6)	C33—C35	1.509 (6)
C14—C16	1.529 (6)	C33—H33	0.9800
C14—H14	0.9800	C34—H34A	0.9600
C15—H15A	0.9600	C34—H34B	0.9600
C15—H15B	0.9600	C34—H34C	0.9600
C15—H15C	0.9600	C35—H35A	0.9600
C16—H16A	0.9600	C35—H35B	0.9600
C16—H16B	0.9600	C35—H35C	0.9600
C16—H16C	0.9600	C36—C38	1.493 (6)
C17—C18	1.490 (6)	C36—C37	1.496 (7)
C17—C19	1.508 (7)	C36—H36	0.9800
C17—H17	0.9800	C37—H37A	0.9600
C18—H18A	0.9600	C37—H37B	0.9600
C18—H18B	0.9600	C37—H37C	0.9600
C18—H18C	0.9600	C38—H38A	0.9600
C19—H19A	0.9600	C38—H38B	0.9600
C19—H19B	0.9600	C38—H38C	0.9600
C19—H19C	0.9600	O1—H1	0.8200
C20—C25	1.387 (4)	O2—H2A	0.8200
			
C2—C1—C6	119.3 (3)	C21—C20—C26	121.5 (2)
C2—C1—C7	119.6 (2)	O2—C21—C22	118.5 (3)
C6—C1—C7	121.0 (3)	O2—C21—C20	121.8 (3)
C3—C2—C1	120.0 (3)	C22—C21—C20	119.7 (3)
C3—C2—H2	120.0	C23—C22—C21	120.4 (3)
C1—C2—H2	120.0	C23—C22—H22	119.8
C2—C3—C4	120.8 (3)	C21—C22—H22	119.8
C2—C3—I1	119.9 (2)	C22—C23—C24	120.6 (3)
C4—C3—I1	119.3 (2)	C22—C23—H23	119.7
C5—C4—C3	120.0 (3)	C24—C23—H23	119.7
C5—C4—H4	120.0	C25—C24—C23	119.3 (3)
C3—C4—H4	120.0	C25—C24—I2	119.8 (2)
C4—C5—C6	120.8 (3)	C23—C24—I2	120.9 (2)
C4—C5—H5	119.6	C24—C25—C20	121.1 (3)
C6—C5—H5	119.6	C24—C25—H25	119.4
O1—C6—C5	118.9 (3)	C20—C25—H25	119.4
O1—C6—C1	122.1 (3)	N2—C26—C20	122.2 (2)
C5—C6—C1	119.0 (3)	N2—C26—H26	118.9
N1—C7—C1	122.3 (2)	C20—C26—H26	118.9
N1—C7—H7	118.9	C32—C27—C28	122.0 (2)
C1—C7—H7	118.9	C32—C27—N2	118.2 (2)
C13—C8—C9	122.1 (3)	C28—C27—N2	119.8 (2)
C13—C8—N1	117.8 (3)	C29—C28—C27	117.3 (3)
C9—C8—N1	120.0 (3)	C29—C28—C36	120.2 (3)
C10—C9—C8	117.6 (3)	C27—C28—C36	122.5 (2)
C10—C9—C17	120.4 (3)	C30—C29—C28	121.6 (3)
C8—C9—C17	122.0 (3)	C30—C29—H29	119.2
C11—C10—C9	121.3 (3)	C28—C29—H29	119.2
C11—C10—H10	119.4	C31—C30—C29	120.0 (3)
C9—C10—H10	119.4	C31—C30—H30	120.0
C10—C11—C12	120.3 (3)	C29—C30—H30	120.0
C10—C11—H11	119.9	C30—C31—C32	121.4 (3)
C12—C11—H11	119.9	C30—C31—H31	119.3
C11—C12—C13	121.7 (4)	C32—C31—H31	119.3
C11—C12—H12	119.2	C27—C32—C31	117.6 (3)
C13—C12—H12	119.2	C27—C32—C33	121.9 (3)
C12—C13—C8	117.1 (3)	C31—C32—C33	120.4 (3)
C12—C13—C14	121.1 (3)	C34—C33—C35	111.9 (4)
C8—C13—C14	121.7 (3)	C34—C33—C32	111.7 (3)
C15—C14—C13	110.6 (3)	C35—C33—C32	112.3 (4)
C15—C14—C16	110.0 (4)	C34—C33—H33	106.8
C13—C14—C16	113.4 (4)	C35—C33—H33	106.8
C15—C14—H14	107.5	C32—C33—H33	106.8
C13—C14—H14	107.5	C33—C34—H34A	109.5
C16—C14—H14	107.5	C33—C34—H34B	109.5
C14—C15—H15A	109.5	H34A—C34—H34B	109.5
C14—C15—H15B	109.5	C33—C34—H34C	109.5
H15A—C15—H15B	109.5	H34A—C34—H34C	109.5
C14—C15—H15C	109.5	H34B—C34—H34C	109.5
H15A—C15—H15C	109.5	C33—C35—H35A	109.5
H15B—C15—H15C	109.5	C33—C35—H35B	109.5
C14—C16—H16A	109.5	H35A—C35—H35B	109.5
C14—C16—H16B	109.5	C33—C35—H35C	109.5
H16A—C16—H16B	109.5	H35A—C35—H35C	109.5
C14—C16—H16C	109.5	H35B—C35—H35C	109.5
H16A—C16—H16C	109.5	C38—C36—C37	109.8 (4)
H16B—C16—H16C	109.5	C38—C36—C28	112.7 (3)
C18—C17—C19	110.0 (4)	C37—C36—C28	111.3 (4)
C18—C17—C9	111.8 (3)	C38—C36—H36	107.6
C19—C17—C9	112.4 (4)	C37—C36—H36	107.6
C18—C17—H17	107.5	C28—C36—H36	107.6
C19—C17—H17	107.5	C36—C37—H37A	109.5
C9—C17—H17	107.5	C36—C37—H37B	109.5
C17—C18—H18A	109.5	H37A—C37—H37B	109.5
C17—C18—H18B	109.5	C36—C37—H37C	109.5
H18A—C18—H18B	109.5	H37A—C37—H37C	109.5
C17—C18—H18C	109.5	H37B—C37—H37C	109.5
H18A—C18—H18C	109.5	C36—C38—H38A	109.5
H18B—C18—H18C	109.5	C36—C38—H38B	109.5
C17—C19—H19A	109.5	H38A—C38—H38B	109.5
C17—C19—H19B	109.5	C36—C38—H38C	109.5
H19A—C19—H19B	109.5	H38A—C38—H38C	109.5
C17—C19—H19C	109.5	H38B—C38—H38C	109.5
H19A—C19—H19C	109.5	C7—N1—C8	121.0 (2)
H19B—C19—H19C	109.5	C26—N2—C27	119.4 (2)
C25—C20—C21	118.9 (2)	C6—O1—H1	109.5
C25—C20—C26	119.5 (2)	C21—O2—H2A	109.5
			
C6—C1—C2—C3	1.5 (4)	C26—C20—C21—C22	−179.2 (3)
C7—C1—C2—C3	−176.8 (3)	O2—C21—C22—C23	−177.4 (3)
C1—C2—C3—C4	−1.7 (5)	C20—C21—C22—C23	2.7 (5)
C1—C2—C3—I1	175.6 (2)	C21—C22—C23—C24	−1.3 (5)
C2—C3—C4—C5	0.1 (5)	C22—C23—C24—C25	−0.9 (5)
I1—C3—C4—C5	−177.2 (3)	C22—C23—C24—I2	176.8 (3)
C3—C4—C5—C6	1.7 (5)	C23—C24—C25—C20	1.7 (4)
C4—C5—C6—O1	177.4 (3)	I2—C24—C25—C20	−176.0 (2)
C4—C5—C6—C1	−1.8 (5)	C21—C20—C25—C24	−0.3 (4)
C2—C1—C6—O1	−179.0 (3)	C26—C20—C25—C24	177.1 (2)
C7—C1—C6—O1	−0.7 (5)	C25—C20—C26—N2	−176.7 (3)
C2—C1—C6—C5	0.2 (4)	C21—C20—C26—N2	0.6 (4)
C7—C1—C6—C5	178.5 (3)	C32—C27—C28—C29	2.1 (4)
C2—C1—C7—N1	176.9 (3)	N2—C27—C28—C29	179.7 (3)
C6—C1—C7—N1	−1.3 (4)	C32—C27—C28—C36	−178.1 (3)
C13—C8—C9—C10	−1.1 (5)	N2—C27—C28—C36	−0.6 (4)
N1—C8—C9—C10	−176.6 (3)	C27—C28—C29—C30	0.2 (5)
C13—C8—C9—C17	178.6 (3)	C36—C28—C29—C30	−179.5 (3)
N1—C8—C9—C17	3.0 (4)	C28—C29—C30—C31	−1.9 (6)
C8—C9—C10—C11	0.0 (6)	C29—C30—C31—C32	1.4 (6)
C17—C9—C10—C11	−179.7 (4)	C28—C27—C32—C31	−2.6 (4)
C9—C10—C11—C12	0.1 (7)	N2—C27—C32—C31	179.8 (3)
C10—C11—C12—C13	0.9 (7)	C28—C27—C32—C33	179.3 (3)
C11—C12—C13—C8	−2.0 (6)	N2—C27—C32—C33	1.7 (4)
C11—C12—C13—C14	−179.9 (4)	C30—C31—C32—C27	0.8 (5)
C9—C8—C13—C12	2.1 (5)	C30—C31—C32—C33	178.9 (3)
N1—C8—C13—C12	177.7 (3)	C27—C32—C33—C34	103.9 (4)
C9—C8—C13—C14	180.0 (3)	C31—C32—C33—C34	−74.2 (5)
N1—C8—C13—C14	−4.4 (4)	C27—C32—C33—C35	−129.4 (5)
C12—C13—C14—C15	77.3 (5)	C31—C32—C33—C35	52.5 (5)
C8—C13—C14—C15	−100.5 (4)	C29—C28—C36—C38	51.6 (5)
C12—C13—C14—C16	−46.8 (5)	C27—C28—C36—C38	−128.2 (4)
C8—C13—C14—C16	135.3 (4)	C29—C28—C36—C37	−72.3 (5)
C10—C9—C17—C18	66.9 (5)	C27—C28—C36—C37	107.9 (5)
C8—C9—C17—C18	−112.8 (4)	C1—C7—N1—C8	−177.2 (3)
C10—C9—C17—C19	−57.3 (5)	C13—C8—N1—C7	99.1 (3)
C8—C9—C17—C19	123.0 (5)	C9—C8—N1—C7	−85.2 (3)
C25—C20—C21—O2	178.3 (3)	C20—C26—N2—C27	175.8 (2)
C26—C20—C21—O2	0.9 (5)	C32—C27—N2—C26	−95.2 (3)
C25—C20—C21—C22	−1.9 (4)	C28—C27—N2—C26	87.2 (3)

### Hydrogen-bond geometry (Å, º)

Cg2 is the centroid of the C8–C13 ring.

**Table:**

*D*—H···*A*	*D*—H	H···*A*	*D*···*A*	*D*—H···*A*
O1—H1···N1	0.82	1.88	2.606 (3)	147
O2—H2*A*···N2	0.82	1.91	2.617 (3)	143
C16—H16*A*···*Cg*2^i^	0.96	2.91	3.785 (5)	153

Symmetry code: (i) *x*−1, *y*, *z*.
